# Modeling Morality in 3‐D: Decision‐Making, Judgment, and Inference

**DOI:** 10.1111/tops.12382

**Published:** 2018-09-14

**Authors:** Hongbo Yu, Jenifer Z. Siegel, Molly J. Crockett

**Affiliations:** ^1^ Department of Experimental Psychology University of Oxford; ^2^ Department of Psychology Yale University

**Keywords:** Moral cognition, Moral decision‐making, Moral judgment, Moral inference, Computational models, Harm aversion

## Abstract

Humans face a fundamental challenge of how to balance selfish interests against moral considerations. Such trade‐offs are implicit in moral *decisions* about what to do; *judgments* of whether an action is morally right or wrong; and *inferences* about the moral character of others. To date, these three dimensions of moral cognition–decision‐making, judgment, and inference–have been studied largely independently, using very different experimental paradigms. However, important aspects of moral cognition occur at the intersection of multiple dimensions; for instance, moral hypocrisy can be conceived as a disconnect between moral decisions and moral judgments. Here we describe the advantages of investigating these three dimensions of moral cognition within a single computational framework. A core component of this framework is *harm aversion*, a moral sentiment defined as a distaste for harming others. The framework integrates economic utility models of harm aversion with Bayesian reinforcement learning models describing beliefs about others’ harm aversion. We show how this framework can provide novel insights into the mechanisms of moral decision‐making, judgment, and inference.

## Abstract 2

The authors explore the interfaces between different dimensions of moral cognition, bridging economic, Bayesian and reinforcement learning perspectives. The human aversion to harming others cuts across these different interfaces, influencing decisions, judgments, and inferences about morality.

## 1 Introduction

**Figure 1:**
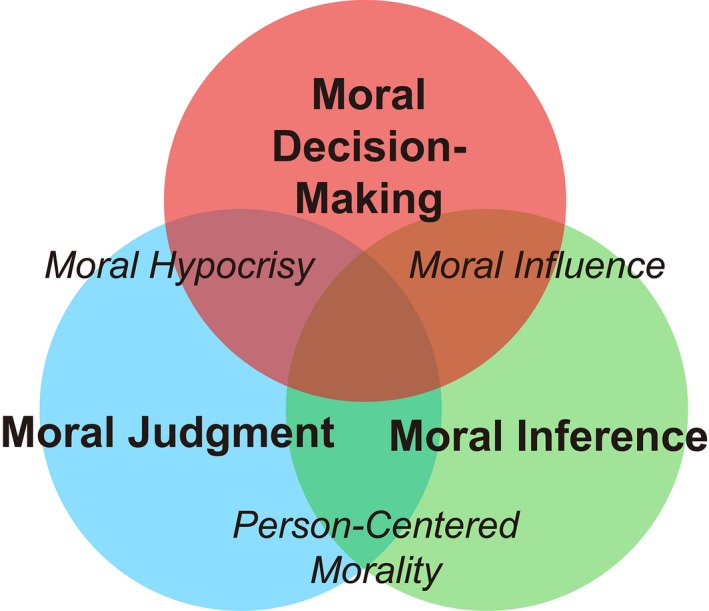
Three dimensions of moral cognition. Most moral cognition research investigates one of three dimensions: moral decision‐making, moral judgment, or moral inference. Important phenomena lie at the intersection of two or more dimensions. For example, moral hypocrisy can be conceptualized as a disconnect between moral decision‐making and moral judgment, where hypocrites judge others harshly for the same decisions they make themselves; in moral influence, inferences about the moral character of others shape one's own moral decisions; and work on person‐centered morality demonstrates that inferences about moral character spill over into moral judgments of individual actions.

On December 14, 2012, Sandy Hook Elementary School teacher Victoria Soto “threw herself in front of her first‐grade students” to protect them from a gunman attacking the school (Planas, 2012). This tragic story illustrates three key dimensions of moral cognition: Soto made a *moral decision* to put her own life in danger to protect her students; you, the reader, probably made a *moral judgment* about whether Soto did the right thing; and from there you probably made a further *moral inference* about what kind of person Soto is in general. Research in moral psychology has traditionally investigated moral cognition along these same three dimensions (Fig. 1):

1. Moral decision‐making: how people make decisions that affect the welfare of others (e.g., Batson, Duncan, Ackerman, Buckley, & Birch, 1981; Batson, Fultz, & Schoenrade, 1987; FeldmanHall, Dalgleish, Evans, & Mobbs, 2015; Gao et al., 2018; Garrett, Lazzaro, Ariely, & Sharot, 2016; Greene & Paxton, 2009; Hsu, Anen, & Quartz, 2008; Koenigs et al., 2007; Rand, Greene, & Nowak, 2012; Sáez et al., 2015: Shalvi, Gino, Barkan, & Ayal, 2015; Zhu et al., 2014).
2. Moral judgment: how people make judgments about the moral appropriateness of actions and assign blame and punishment, or praise and reward (e.g., Baron, 2014; Greene, Sommerville, Nystrom, Darley, & Cohen, 2001; Malle, Guglielmo, & Monroe, 2014; Schein & Gray, 2014, 2015, 2018; Shenhav & Greene, 2010; Wojciszke, Parzuchowski, & Bocian, 2015; Young & Saxe, 2008; Young et al., 2010).
3. Moral inference: how people form beliefs about the moral character of agents based on observations of morally relevant behaviors (e.g., Alicke & Zell, 2009; Bostyn & Roets, 2017; Behrens, Hunt, Woolrich, & Rushworth, 2008; Diaconescu et al., 2014; Everett, Pizarro, & Crockett, 2016; Hackel, Doll, & Amodio, 2015; Giner‐Sorolla & Chapman, 2017; Kliemann, Young, Scholz, & Saxe, 2008; Kleiman‐Weiner, Saxe, & Tenenbaum, 2017; Knobe, 2010; Nadler, 2012).

To date, these three dimensions have been investigated mostly independently, usually by different researchers using very different experimental paradigms. For example, moral decision‐making has typically been studied with tasks involving incentivized choices affecting the welfare of others; moral judgment has typically been studied using hypothetical “dilemma” scenarios; and moral inference has typically been studied using narrative descriptions of moral/immoral behaviors. Here, we advocate for investigating moral decision‐making, judgment, and inference within the same experimental framework that incorporates computational models of cognitive processes. We propose that this approach can advance the study of moral cognition in several ways. First, it can reveal common computations underlying moral decision‐making, judgment, and inference. Second, it can facilitate the investigation of many important moral phenomena that involve intersections across dimensions, such as moral hypocrisy, moral influence, and person‐centered moral judgments (Fig. 1).

In the following, we introduce an example experimental framework that can be used to concurrently investigate three dimensions of moral cognition in the domain of harm. This framework incorporates computational models that describe how external features of a moral problem (e.g., harm, benefit, causation, intention, character, etc.) can be transformed into an internal utility, and how this utility is used to guide moral decision‐making, judgment, and inference (Crockett, 2016). Formal model comparison procedures are used to compare the predictive power of different models that make different assumptions about how people make decisions, judgments, and inferences, testing the ability of a hypothesized set of cognitive processes to account for the entire set of choices people make as well as patterns of brain activity (Daw, 2011; Fehr & Krajbich, 2014; Hutcherson et al., 2015; Konovalov et al., 2018; Krakauer et al., 2017; Love, 2015 O'Doherty, Hampton, & Kim, 2007). Using this approach, it may be possible to reveal common computations in moral decision‐making, judgment, and inference by examining, for example whether similar models can describe behavior along different dimensions; whether individual differences in one dimension predict individual differences in other dimensions; and whether there are similar neural processes underlying different computations across dimensions. We provide examples of such evidence in the following sections.

Thereafter, we describe how this approach may be able to illuminate the nature of complex moral phenomena that lie at the boundary of two areas of moral cognition: person‐centered morality (Alicke, Mandel, Hilton, Gerstenberg, & Lagnado, 2015; Knobe, 2010; Tannenbaum, Uhlmann, & Diermeier, 2011; Uhlmann, Pizarro, & Diermeier, 2015), moral hypocrisy (Batson, Kobrynowicz, Dinnerstein, Kampf, & Wilson, 1997; Gino, Norton, & Weber, 2016; Graham, Meindl, Koleva, Iyer, & Johnson, 2015; Sharma, Mazar, Alter, & Ariely, 2014; Szabados & Soifer, 2004), and moral influence (Bandura, 1969; Cialdini & Goldstein, 2004; Hoffman, 1970; Gino, Ayal, & Ariely, 2009; Macaulay & Berkowitz, 1970; Staub, 1971). Although the illustrative examples provided in this paper are specific to just one domain of morality (i.e., harm), the approach we describe can potentially be applied to other moral domains as well.

## 2 Harm aversion as a core component of moral cognition across dimensions

Our framework adopts the view that the computation of utility or value of a particular action for oneself, other individuals, and/or society comprises a core component of moral cognition (Bartels, Bauman, Cushman, Pizarro, & McGraw, 2015; Crockett, 2013, 2016; Cushman, 2013; Shenhav & Greene, 2010) and is related to the moral philosophy of utilitarianism, which posits that morally right action is the action that produces the most good or utility (e.g., Mill, 1863/1998). We propose that a key subcomponent of utility in moral cognition is *harm aversion*: a moral sentiment defined as a distaste for harming others. Although it is still debated whether harm is the essence of morality (Gray, Young, & Waytz, 2012; Schein & Gray, 2018) or just one of several moral “foundations” (Graham et al., 2011), it is widely acknowledged that avoiding harm to others is a universal moral principle (Gert, 2004; Keane, 2015) and comprises the majority of moral experiences in daily life (Hofmann, Wisneski, Brandt, & Skitka, 2014).

Studies of harm aversion in moral judgment, decision‐making, and inference have typically relied on very different methods. Most studies of moral judgment rely on hypothetical scenarios, such as the classic “trolley problem” where participants are asked if it's acceptable to push a large man off a bridge to stop a trolley from running over several track workers (e.g., Greene et al., 2001). Meanwhile, studies of moral decision‐making generally ask participants to make choices in the laboratory that have actual consequences for themselves and others, such as trading off money for oneself against electric shocks to others (e.g., FeldmanHall et al., 2012; Crockett, Kurth‐Nelson, Siegel, Dayan, & Dolan, 2014). Finally, research on moral inference typically asks participants to form impressions of others based on descriptions of morally relevant behaviors, such as performing good deeds or committing crimes (e.g., Goodwin, Piazza, & Rozin, 2014; Uhlmann et al., 2015).

The methods used to study each of these dimensions of harm‐based moral cognition have been developed for good reasons, but using diverse paradigms to measure different dimensions may hinder the identification of common computational processes that operate across multiple dimensions. If such common computations exist, individual variability in one dimension of moral cognition might predict variability along other dimensions. For example, individuals who are highly harm averse in their moral decisions may also be highly harm averse in their moral judgments. It is difficult to address this question definitively using different paradigms to measure different dimensions. For example, it is difficult to know whether the same kind of harm aversion motivates the judgment that one should not push people off bridges as well as the decision to avoid delivering electric shocks to others. If one observes a positive correlation between harm aversion in trolley judgments and shock decisions, it is difficult to attribute this relationship to a common computational process because trolley problems do not explicitly measure computation; if no correlation is observed, this may be due to the large differences between paradigms. One way to make meaningful comparisons between different dimensions of moral cognition is to develop a paradigm that can simultaneously interrogate the computational processes underlying moral decision‐making, judgment, and inference within the same setting. This approach makes it possible to begin testing the hypothesis that different dimensions of moral cognition are built upon a few basic computations, such as computing utility by trading off costs and benefits to oneself against costs and benefits to others (see also Jara‐Ettinger, Gweon, Schulz, & Tenenbaum, 2016).

### 2.1 Modeling harm aversion in moral decision‐making

**Figure 2:**
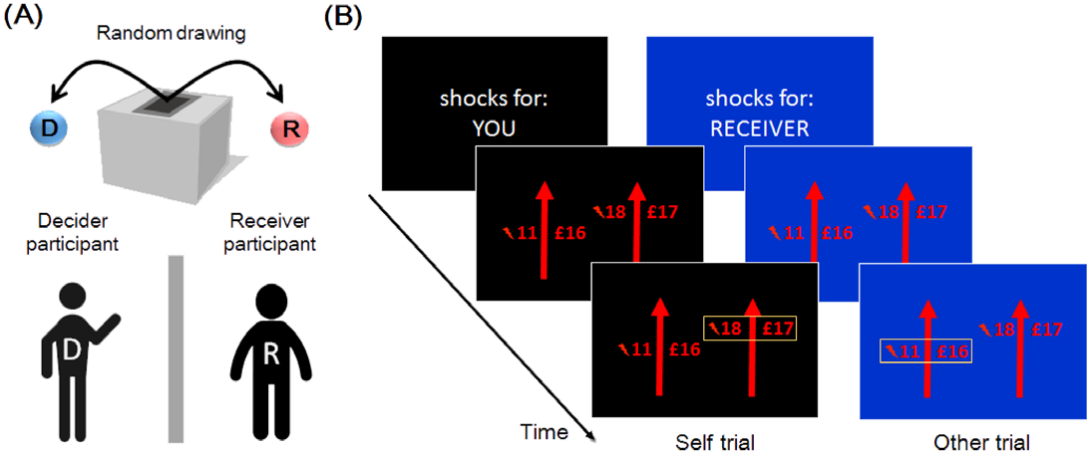
Moral decision task. (A) Participants are randomly assigned to the roles of Decider and Receiver. (B) Deciders make a series of choices where they are asked to trade‐off money for themselves against pain to either themselves (Self trial) or the Receiver (Other trial).

Recently, we examined harm aversion in moral decision‐making by investigating how people trade off money for themselves against pain for themselves and others (Crockett et al., 2014, 2015; Crockett, Siegel, Kurth‐Nelson, Dayan, & Dolan, 2017; Fig. 2). In this paradigm, participants (“Decider”) make choices between different amounts of money and different numbers of painful electric shocks directed toward either themselves or an anonymous other person (“Receiver”). Computational models formally quantify the relative values people ascribe to pain for themselves and others, and how those values are transformed into choices.

The valuation of harmful actions affecting oneself and others can be described by different models that contain different parameters or have different ways of integrating the values of pain and money. Crockett et al. (2014) compared a number of models and found participants’ decisions were best described by a model that contained independent parameters describing harm aversion for self and others:

(1a) $$V\left(\mathrm{harm}\right)=\left(1-\kappa \right)\Delta m-\kappa \Delta s$$

(1b) $$P\left(\mathrm{choose}\;\mathrm{harm}\right)=\frac{1}{1+e^{-\beta V\left(\mathrm{harm}\right)}}$$

The model relates objective features of the choice options (here, amounts of money difference, Δm, and shock difference, Δs) to their underlying subjective values (i.e., *V*(harm)). A softmax function (Eq. 1b) transforms the relative subjective value of choosing the harmful option into a probability of that choice, where the parameter β defines the steepness of the slope in the softmax function: Larger β corresponds to a steeper slope and indicates more deterministic choice preference. The money and shock terms are scaled by a harm aversion parameter (*κ*) that quantifies the exchange rate between money and pain and takes on different values for pain to self and others. Strikingly, across several studies harm aversion for others was consistently greater on average than harm aversion for self (Crockett et al., 2014, 2015, 2017), an effect that has been replicated by an independent research group using a different paradigm (Volz, Welborn, Gobel, Gazzaniga, & Grafton, 2017). People were willing to pay more to prevent shocks to others than to themselves, and required more compensation to increase shocks to others than themselves; that is, their behavior was “hyperaltruistic” (Kitcher, 1993). This pattern of choice cannot be readily explained by classic theories of empathy or social preferences, which posit that people value others’ welfare no more (and often much less) than their own welfare (Batson et al., 1981; Engel, 2011; Singer et al., 2004). However, hyperaltruism is consistent with work in moral psychology suggesting people experience aversive feelings (e.g., guilt or fear of blame) when causing bad outcomes, especially when those outcomes affect others (Cushman, Gray, Gaffey, & Mendes, 2012a; Ritov & Baron, 1990). These aversive feelings, or expectations of them, might degrade the value of actions that harm others (Baumeister, Stillwell, & Heatherton, 1994; Chang, Smith, Dufwenberg, & Sanfey, 2011; Charness & Dufwenberg, 2006; Lewis, 1971; Yu, Hu, Hu, & Zhou, 2014).

There are at least two possible mechanistic explanations for hyperaltruism. First, people may compute the value of others’ pain as more aversive than their own pain. Alternatively, money gained immorally (i.e., via harming others) may be subjectively less valuable than money gained from harming only oneself, perhaps due to discomfort, guilt, or anticipation of being blamed or judged. Because the harm aversion parameter in the model represents an exchange rate between money and pain, the parameter estimates alone do not straightforwardly reveal the underlying cognitive process. However, because harm aversion is the output of a computational process integrating the values of money and pain, it is reasonable to hypothesize that harm aversion might covary with neural responses to money, pain, or both. Thus, by combining the model with fMRI it is possible to interrogate how the brain represents profit and pain during moral decision‐making, and whether individual differences in neural responses to profit or pain track with individual differences in hyperaltruism. This approach sidesteps the necessity for informal reverse inference because the model makes trial‐by‐trial predictions about neural responses to profit, pain, value, and so on (Behrens, Hunt, & Rushworth, 2009). If hyperaltruism arises from an increased weighting of others’ pain relative to one's own, then pain‐sensitive brain regions should show relatively increased responses to others’ pain, to the extent people are hyperaltruistic. Meanwhile if hyperaltruism arises from a reduced valuation of ill‐gotten gains relative to profits gained morally, then profit‐sensitive brain regions should show relatively reduced responses to ill‐gotten gains, to the extent people are hyperaltruistic.

**Figure 3:**
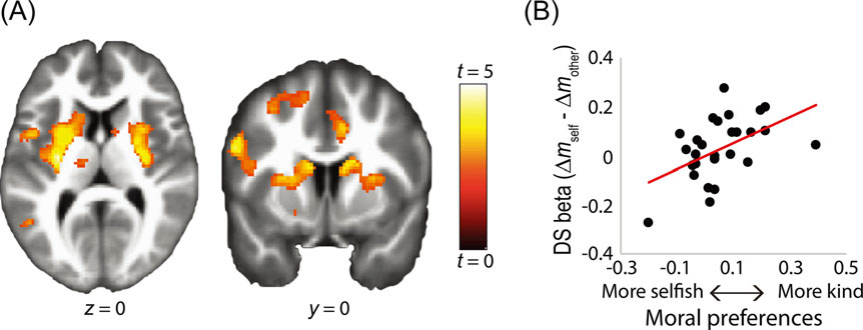
The neural basis of hyperaltruism. (A and B) In bilateral DS, reduced responses to profits gained from harming others correlated with hyperaltruism (*κ*_other_ − *κ*_self_). Figure adapted from Crockett et al. (2017).

A recent neuroimaging study (Crockett et al., 2017) found strong evidence for the latter hypothesis. Although the insula and anterior cingulate cortex (ACC) responded to anticipated pain for self (and to a lesser extent for others), individual differences in “empathic” pain responses in these regions did not predict individual differences in hyperaltruism. In fact, there were no brain areas where differential responses to others’ versus own pain correlated with hyperaltruism. Meanwhile, responses in the brain's valuation network, in particular the dorsal striatum (DS), showed reduced responses to money gained from shocking others relative to money gained from shocking self, to the extent that people were hyperaltruistic (Fig. 3). This indicates that moral behavior might arise from a devaluation of profits gained from harming others.

### 2.2 Modeling harm aversion in moral judgment

**Figure 4:**
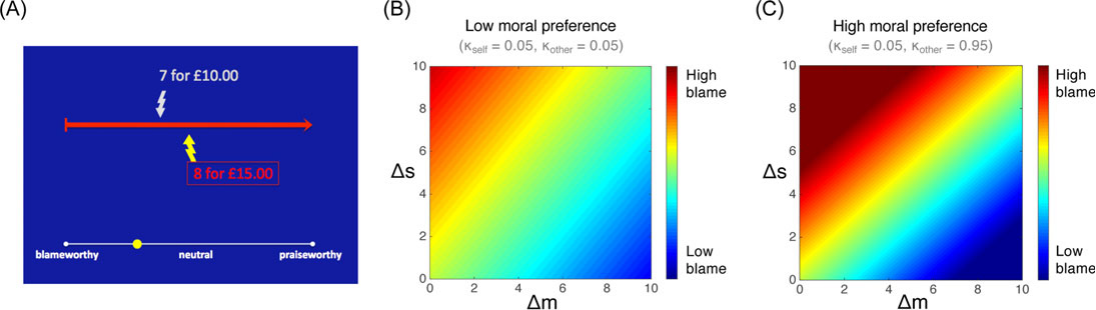
Moral judgment task and model. (A) Participants are presented with decisions that others have made and asked to judge the extent to which those decisions are blameworthy or praiseworthy. (B and C) Heatmaps depicting patterns of moral judgment by participants with low hyperaltruism (B) and high hyperaltruism (C) vary as a function of extra shocks and extra money resulted from choosing the more harmful option. Figure adapted from Crockett et al. (2017).

The moral decision task can be easily modified to investigate moral judgment. In the moral judgment task, instead of deciding whether to profit from inflicting pain on others, participants are presented with decisions that others have made and asked to judge the extent to which those decisions are blameworthy or praiseworthy (Fig. 4A). Computational models can then be built to describe how judgments of blame and praise are sensitive to, for example the amount of pain inflicted, the amount of profit gained, and whether the decision was made actively or passively. Crucially, by asking participants to complete both the moral decision task and the moral judgment task, it is possible to describe how one's own moral preferences are related to moral judgments of others’ behavior within an identical context. In doing so it is possible to address questions such as whether “conscience” in moral decision‐making can be conceptualized as a turning‐inward of moral judgments normally applied to others (Smith, 1759/2002; Freud, 1961), and whether people who are more harm averse in moral decision‐making are also more harm averse in their moral judgments.

In a recent study (Crockett et al., 2017), participants first completed the moral decision task depicted in Fig. 2, through which we could estimate their degree of harm aversion for themselves (*κ*_*s*_) and for others (*κ*_*o*_). Next, participants completed the moral judgment task depicted in Fig. 4A where on each trial they judged the blameworthiness of choices made by another agent. Crucially, in the moral judgment task the differences in shocks (*Δs*) and profit (*Δm*) resulting from the agent's choices were decorrelated across trials. This not only enabled us to estimate the independent contributions of pain and profits to moral judgments, but also how people's own harm aversion influences their reliance on pain and profit in making moral judgments. Thus, in our moral judgment model, trial‐by‐trial blame judgments were regressed against trial features (*Δs* and *Δm*), individuals' own harm aversion (*κ*_*s*_ and *κ*_*o*_), and their interactions:

(2) $$\begin{array}{cc} Blame_{t}=\beta_{0}+\beta_{1}\Delta m+\beta_{2}\Delta s+\beta_{3}\Delta m\kappa_{o}+\beta_{4}\Delta m\kappa_{s} & \\ \;\;\;\;\;\;\;\;+\beta_{5}\Delta s\kappa_{o}+\beta_{6}\Delta s\kappa_{s}+\beta_{7}\Delta m\kappa_{s}\kappa_{o}+\beta_{8}\Delta s\kappa_{s}\kappa_{o} \end{array}$$

The regression revealed several key findings. First, moral judgments of blameworthiness involved very similar computations as in moral decision‐making. Blame was negatively correlated with the additional profit (*Δm*) caused by choosing the more harmful option and positively correlated with the additional pain (*Δs*) caused by choosing the more harmful option. This indicates that although blame is sensitive to harmful outcomes (Crockett, Clark, Hauser, & Robbins, 2010; Cushman, Young, & Hauser, 2006), the profits gained seem to justify harm at least partially, consistent with work using hypothetical scenarios (Xie, Yu, Zhou, Sedikides, & Vohs, 2014). Notably, these results show that money and pain exerted opposing effects on the computation of blame in moral judgments, just as they exerted opposing effects on the computation of value in moral decision‐making.

Second, participants’ own harm aversion preferences (*κ*_*s*_ and *κ*_*o*_) modulated the influence of profit and pain on blame, such that the participants who themselves were more averse to causing pain to others (higher *κ*_*o*_) made more extreme blame judgments and cared more about harm and less about profit in making blame judgments (Fig. 4B and C). In other words, people who were more harm averse in moral decision‐making also showed a stronger influence of harm on moral judgments.

In addition, data from the moral judgment task allowed us to probe a long‐standing theory about the nature of moral preferences (Smith, 1759/2002). Crockett et al. (2017) hypothesized that the devaluation of ill‐gotten gains could arise from a top‐down modulation of value‐sensitive regions by lateral prefrontal cortical (LPFC) regions involved in representing moral norms (Buckholtz, 2015), similar to the way long‐term health goals represented in LPFC modulate the value of unhealthy foods (Hare, Camerer, & Rangel, 2009). That is, LPFC may represent shared norms about what is morally appropriate and modulate the value of profits to the extent they are gained via blameworthy actions. This account predicts that LPFC activity during moral decision‐making tracks the blameworthiness of harmful actions. To test this account, we used our model of moral judgment (Eq. 2) to construct an individualized “blame regressor” for each participant who completed the moral decision task in the fMRI scanner and probed the relationship between LPFC activity at time of choice and blameworthiness of each choice. Note that the participants in the fMRI scanner never made blame judgments themselves. Rather, our goal was to predict their LPFC activity based on how other participants judged the blameworthiness of harmful actions. Such analysis was possible because both moral decision‐making and moral judgment were measured quantitatively within the same experimental paradigm and computational framework. If brain activity during moral decision‐making could be predicted by a model of moral judgments, this would provide support for the hypothesis that common computations underlie moral judgment and moral decision‐making.

Remarkably, LPFC activity at time of choice was indeed significantly correlated with other people's moral judgments of the blameworthiness of choosing the profitable but harmful option. Its response was strongest on those trials where profiting through harm was judged to be the most blameworthy by others–prototypically when the harmful action inflicted a large amount of pain for a tiny amount of profit. This finding captures the essence of how moral norms operate: When we make moral decisions, we simulate how an “impartial spectator” would judge us for violating the norm. Finally, supporting a moral devaluation account, we found that during moral decisions, LPFC was functionally connected with the same region of striatum that showed a reduced response to ill‐gotten gains. These findings suggest that rather than restraining self‐interest via inhibitory control processes, moral norms modulate the value of harmful actions. In other words, it's not that people are constantly tempted to harm others for their own benefit and have to override these temptations. Rather, moral norms make selfish actions less tempting in the first place.

While the above paradigm focused on moral judgments by disinterested third‐parties, it can also be adapted to investigate moral judgments and affective responses of second‐party targets of moral decisions. For example, in a recent fMRI study of gratitude (Yu, Gao, Zhou, & Zhou, 2018), participants received costly help from a co‐player, who sacrificed their own profits to reduce participants’ pain. Gratitude was well predicted by a model that integrated the co‐player's sacrificed profits and participants’ pain reduction in a way remarkably similar to the models of moral decision‐making and judgment described above. Specifically, gratitude was positively related to pain reduction and the co‐player's sacrificed profits. Moreover, trial‐by‐trial gratitude as predicted by the model was correlated with activity in vmPFC, just as trial‐by‐trial estimates of subjective value in moral decision‐making correlated with responses in this region (Crockett et al., 2017). Finally, results indicated that gratitude was more sensitive to the co‐player's sacrificed profits than pain reduction just as moral decisions were explained better by neural responses to profits than pain in a similar setting. Together these findings suggest that basic computations of utility integrating costs and benefits for self and others may contribute to multiple dimensions of moral cognition, including decisions to harm others, evaluations of blame and praise, and feelings of gratitude.

### 2.3 Modeling harm aversion in moral inference

Moral evaluation does not stop at the level of judging single events (e.g., right vs. wrong), but very often proceeds from there to making inferences about the moral character of agents (e.g., good vs. evil), a process that has been referred to as moral inference (cf. Everett et al., 2016, 2018; Helzer & Critcher, 2018; Knobe, 2010; Uhlmann et al., 2015). Accurately inferring the moral character of others helps predict their behaviors (Fiske, Cuddy, & Glick, 2007). Some forms of moral inference might be understood as a dynamic, evidence‐accumulation process. However, the cognitive mechanisms through which people form and update beliefs about the moral character of others are not well understood.

**Figure 5:**
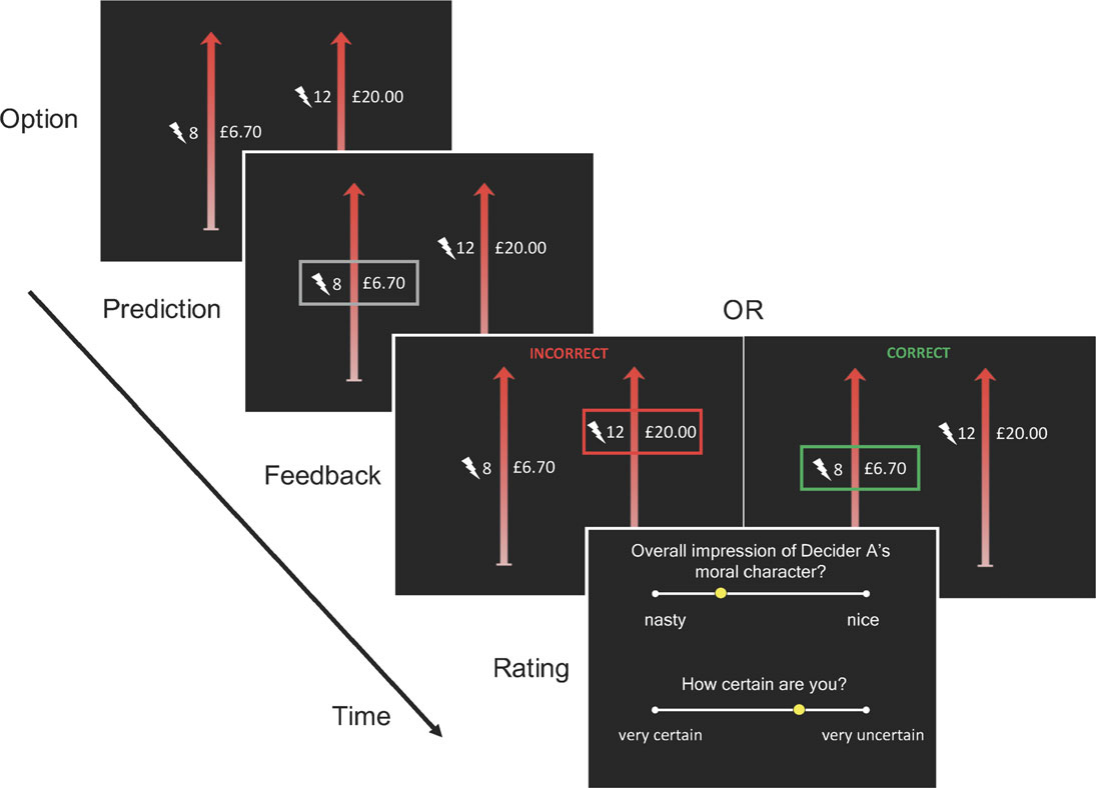
Moral inference task. On every trial the agent chose between two options: more money for themselves plus more shocks for an anonymous receiver, or less money for themselves plus fewer shocks for the receiver. Participants predicted which option the agent would choose and subsequently received feedback about their accuracy. Every a few trials, participants provided their subjective impression of the agent's character on a scale ranging from “nasty” to “nice,” as well as their uncertainty of that impression.

We adapted our experimental paradigm to study the computational processes guiding moral inference (Fig. 5). In particular, we were interested in testing the possibility that people would successfully predict the moral decisions of other agents by accurately inferring their level of harm aversion and updating beliefs about harm aversion in accordance with Bayes’ rule, which would be consistent with recent work on inference in the domains of perception (Ma, Beck, Latham, & Pouget, 2006), economic value (Schwartenbeck et al., 2015), and social intention (Behrens et al., 2009; Diaconescu et al., 2014, 2014). In the moral inference task, instead of deciding whether to profit from inflicting pain on others, participants are asked to predict the decisions that other agents will make (Fig. 5). Computational models describe how beliefs about the harm aversion of other agents develop over time. By examining moral inference and moral decision‐making within the same framework, it is possible to test whether people use similar computations to make moral decisions themselves and predict the moral decisions of others.

In a series of studies (Siegel, Mathys, Rutledge, & Crockett, in press), participants predicted sequences of choices in the moral decision task made by two agents. Periodically, participants provided their subjective impression of the agent's character on a scale ranging from “nasty” to “nice”, as well as their uncertainty of that impression. The two agents differed substantially in their level of harm aversion for others (κ_o_): The “good” agent required more than five times the compensation per shock to the receiver than the “bad” agent. We tested the hypothesis that participants’ predictions about the agents’ choices reflected their estimates of the agents’ harm aversion by fitting their trial‐by‐trial predictions with a computational model that combined our utility model of moral decision‐making (Crockett et al., 2014) with a type of Bayesian learning model, the Hierarchical Gaussian Filter (HGF; Mathys, Daunizeau, Friston, & Stephan, 2011). The HGF provides a mathematical account of how people update beliefs about hidden states based on an integration of prior beliefs about the hidden state and new observations. In our moral inference setting, beliefs are defined as probability distributions over a range of levels of harm aversion, and people update their beliefs about another agent's harm aversion based on observing that agent's decisions trading off money for themselves against pain for another. Our moral inference model specifies that participants make predictions about another agent's choices by passing their current belief about that agent's harm aversion (μ) through the utility model that describes choices in this same setting (Crockett et al., 2014; see Eq. 1a):

(3a) $$V_{\mathrm{agent}}\left(\mathrm{harm}\right)=\left(1-\mu \right)\Delta m-\mu \Delta s.$$

(3b) $$P\left(\mathrm{predict}\;\mathrm{harm}\right)=\frac{1}{1+e^{-\beta V\left(\mathrm{harm}\right)}}$$

β is a free parameter that describes how sensitive predictions are to the relative utility of different outcomes, or the prediction noise. When feedback is received (i.e., whether the prediction was correct or incorrect), beliefs are updated in proportion to the uncertainty of the belief, in accordance with Bayes’ rule:

(4) $$\mu_{i}\propto \mu_{i-1}+\sigma_{i-1}\cdot \delta$$

Conceptually, the belief about the agent's harm aversion at trial *i* (μ_*i*_) is updated such that the updated belief is a function of the prior belief (μ_*i*‐1_), plus a prediction error (δ) weighted by the uncertainty of the prior belief (σ_*i*‐1_). This update equation is similar in form to that in classical reinforcement learning models (e.g., Rescorla‐Wagner models), but instead of multiplying the prediction error by a single learning rate, here the prediction error is weighted by the uncertainty of the prior belief, which changes dynamically across trials. In Bayesian inference, this uncertainty indicates how much learning still needs to occur. The model provides, for each participant, a trial‐by‐trial trajectory of belief estimates about each agent's harm aversion (μ); a trajectory of associated uncertainties on those beliefs (σ); and a global estimate of belief volatility (ω) that describes how flexibly participants update their beliefs about each agent's character based on observed decisions. The model explained significant variation in participants’ predictions (capturing 87% of variance for lab studies, and 76% of variance for online studies) and outperformed a simpler Rescorla‐Wagner learning model, which does not allow belief updates to vary in proportion to belief uncertainty.

Two notable findings emerge from the modeling results (Siegel et al., in press). First, the model demonstrates that similar computations are used to make moral decisions oneself, and to predict moral decisions in others. Second, by explicitly modeling the uncertainty and volatility of beliefs, we discovered that beliefs about the harm aversion of bad agents were more uncertain than beliefs about the harm aversion of good agents. As a result, beliefs about bad agents were more volatile than beliefs about good agents, indicating that in response to new information, people more readily revised their impressions about the bad agent than the good agent. These findings suggest a cognitive mechanism that could facilitate forgiveness, which involves changing one's attitudes toward transgressors (Beyens, Yu, Han, Zhang, & Zhou, 2015; Griswold, 2007; McCullough, Pargament, & Thoresen, 2001).

Control experiments showed that this asymmetry in belief updating was not observed when making inferences about low‐skilled and high‐skilled agents’ competence. We adapted the inference paradigm such that participants predicted whether other agents would be able to score a certain number of basketball points in a given amount of time; competence was operationalized as the number of basketball points the agent could score per minute of game play. We found that a similar Bayesian updating model described predictions about basketball scoring as that which described predictions about moral choices, where beliefs about harm aversion were replaced with beliefs about competence. Notably, however, while participants formed more uncertain and volatile beliefs about bad agents’ character, relative to good agents’ character, beliefs about the competence of high‐skill and low‐skill agents were equally uncertain and volatile (Siegel et al., in press). This suggests that our experimental approach can not only be used to compare computational processes across different dimensions of moral cognition but can also shed light on differences between computational processes engaged in moral versus non‐moral cognition.

## 3 Intersecting dimensions of moral cognition

Many important moral phenomena involve more than one dimension of moral cognition. The approach we describe here is most valuable in these cases, as it offers the possibility to quantify cross‐dimension interactions hypothesized to underlie theoretically and practically interesting phenomena, such as person‐centered morality, moral hypocrisy, and moral influence (Fig. 1). In this section, we will briefly discuss how the harm aversion paradigm and computational model outlined above could be utilized to investigate the neurocognitive basis of these phenomena.

### 3.1 Person‐centered morality: Inferences ∩ judgments

Much research on moral judgment has focused predominantly on the evaluation of acts (i.e., act‐centered), singling out features of acts that influence their moral evaluation (Baron, 2014; Malle et al., 2014; Shaver, 2012; Weiner, 1995), including the consequences of the act, the intentions of the actor, and the extent to which the actor caused the consequences (Cushman, 2008; Cushman, Murray, Gordon‐McKeon, Wharton, & Greene, 2012b; Ginther et al., 2016; Karlovac & Darley, 1988; Shaver, 2012; Shultz & Wright, 1985; Shultz, Wright, & Schleifer, 1986; Weiner, 1995). However, in real‐life situations, moral evaluations are often made with the knowledge of the moral character of the agent being evaluated. Recent work on such “person‐centered” evaluations has explored how inferences about an agent's moral character influence moral judgments of that agent's acts (Alicke et al., 2015; Knobe, 2010; Tannenbaum et al., 2011; Uhlmann et al., 2015). Past research has demonstrated significant effects of character inferences on the evaluation of consequences, causation, and blame. We recently applied our computational framework to investigate how inferences about character affect the computation of blame via the evaluation of different aspects of moral acts, such as the consequences of the act and the degree to which the agent is causally responsible for those consequences.

Siegel, Crockett, and Dolan (2017) combined the moral judgment task (described in the previous section) with a manipulation of agents’ moral character. Participants judged the blameworthiness/praiseworthiness of a series of decisions made by two agents with differing moral character. As in the moral inference task, we operationalized the moral character of the agents according to the harm aversion parameter in the decision model, where the “good” agent required more compensation to shock the receiver than the “bad” agent. To manipulate causation, the agent chose the more harmful option either passively (by default) or actively. To manipulate consequences, the agents’ choices resulted in different amounts of profit for themselves and pain for the receiver. Here, because both the moral character of the agents (i.e., degree of harm aversion) and the features of their particular decision (i.e., amount of pain; and money) are quantitatively manipulated within the harm aversion framework, it is possible to demarcate and compare the contributions of person‐level (i.e., moral character) and choice‐level (i.e., money, pain, causation) features to moral judgments.

Results showed an effect of moral inference on the computation of blame: participants weighted the consequences of choices (i.e., profit and pain) more strongly in their judgments of bad agents’ choices than good agents’ choices. Specifically, profits mitigated the blameworthiness of harmful choices, and this effect was larger for the bad than the good agent. Meanwhile, blameworthiness scaled with the amount of pain inflicted, and this effect was also larger for the bad than the good agent. The increased weighting of consequences in judgments of bad agents may reflect enhanced attention toward the behaviors of potentially harmful individuals, the avoidance of whom may have benefits for survival (Tooby & Cosmides, 1992). Future work could usefully adapt Bayesian models of moral inference to interrogate how dynamically evolving impressions of agents’ character shape subsequent moral judgments.

### 3.2 Moral hypocrisy: Decisions ∩ judgments

Moral hypocrisy occurs when people hold themselves to different moral standards than others, and it likely reflects a motivation to appear moral while behaving selfishly (Batson et al., 1997; Gino et al., 2016; Graham et al., 2015; Jordan, Sommers, Bloom, & Rand, 2017; Sharma et al., 2014; Szabados & Soifer, 2004). Researchers have operationalized hypocrisy in two complementary ways. One defines hypocrisy as a discrepancy between judgments and decisions: that is judging a decision to be wrong while nevertheless making that decision oneself (Batson & Thompson, 2001; Batson, Thompson, & Chen, 2002; Batson, Thompson, Seuferling, Whitney, & Strongman, 1999; Batson et al., 1997; Stone, Wiegand, Cooper, & Aronson, 1997). The second defines hypocrisy as a discrepancy between judgments of one's own and others’ behavior, that is judging another person more harshly for transgressing than judging oneself for doing the same thing (Valdesolo & DeSteno, 2007, 2008). Moral hypocrisy is a widespread phenomenon, but its underlying mechanisms are not well understood.

Within our harm aversion framework, the first definition of moral hypocrisy arises at the intersection of moral decision‐making and moral judgment (Fig. 1). Investigating hypocrisy within this framework can illuminate its underlying mechanisms. For example, participants can complete the moral decision task as well as the moral judgment task, and hypocrisy can be defined as a discrepancy between the indifference point for decisions and the indifference point for judgments. Given that moral decision‐making involves a devaluation of ill‐gotten gains via corticostriatal interaction (Crockett et al., 2017), one prediction is that hypocrites would show a reduced coupling between prefrontal regions that represent moral norms and striatal regions that represent the value of one's own actions. In other words, hypocrites may adequately represent moral norms but be unable to translate those norms into moral actions. This would be consistent with reports that criminal psychopaths, who have impaired corticostriatal function (Hosking et al., 2017), show intact moral judgments despite committing moral atrocities (Glenn, Raine, Schug, Young, & Hauser, 2009).

The second definition of hypocrisy can be operationalized within our framework as a discrepancy in the indifference point for judgment of one's own and others’ decisions. One possible explanation for this kind of hypocrisy is readily apparent from our model of moral judgment (Eq. 2), where blame is mitigated by the profitability of harmful actions (Crockett et al., 2017; Siegel et al., 2017). It is well established that people value others’ profits far less strongly than their own (Engel, 2011; Ruff & Fehr, 2014). Thus, it arises naturally from our model that people should blame others more than they blame themselves for the same profitable but harmful action, because profits for others are valued less (and thus would mitigate blame less) than profits for oneself. This account further predicts that those who place a higher value on others’ rewards, that is those who are more generous, should be less hypocritical.

### 3.3 Moral influence: Inferences ∩ decisions

The question, “Can virtue be taught?” (Plato's *Meno*) has intrigued moral philosophers and educators for thousands of years. The effects of role models on prosocial and antisocial behaviors have been extensively studied since the early days of social psychology (Bandura, 1969; Cialdini & Goldstein, 2004; Hoffman, 1970; Macaulay & Berkowitz, 1970; Staub, 1971). For example, in one of Milgram's (1965) experiments, participants were instructed to deliver increasingly painful electric shocks to a receiver. The presence of confederates who refused to increase the shocks discouraged participants from escalating the pain (see also Rosenhan, 1969). More generally, research has shown that the degree of social influence is moderated by several factors, including how much people identify with and like the role model (Abrams & Hogg, 1990; Izuma & Adolphs, 2013), which may be moderated by inferences observers make about the role model's character. Thus, to better understand moral influence, we need a mechanistic account of how observers infer the moral character of role models and how such inferences might influence the observer's own decision‐making.

Within our proposed framework (Fig. 1), moral influence can be understood as an interaction between moral inference and moral decision‐making. By asking participants to complete the moral decision task before and after the moral inference task, it is possible to measure the effect that inferring the moral character of a role model has on one's own moral decisions. Additionally, we can probe how one's own baseline harm aversion moderates the extent of moral influence. In other words, is a morally “better” person (e.g., *κ*_*o*_ **= 0.7) more susceptible to influence than a morally “worse” person (e.g., *κ*_*o*_ **= 0.3)? Does the degree of influence depend on a person's objective or perceived similarity to the model (cf. Han, Kim, Jeong, & Cohen, 2017)? Our framework makes it possible to answer these questions because it offers a platform where the moral preferences of the role model and those of the participants can be precisely parameterized and compared quantitatively.

Understanding how role models influence the moral decisions of other people can help determine how to most effectively leverage the persuasive power of moral exemplars. For example, if the morally “worse” are more susceptible to influence, this would suggest sending role models to high‐risk audiences, such as prisons; whereas if the morally “average” are more susceptible, this would suggest sending role models to more general audiences, such as schools. By jointly testing how the degree of influence depends on participants’ actual similarity with the role model and perceived similarity with the role model, we can further identify channels for influence. For example, if perceived similarity between oneself and moral exemplars enhances susceptibility to influence, this suggests (perhaps counterintuitively) that making people feel they are more moral than they actually are, thus increasing perceived similarity with role models, could facilitate moral change.

## 4 Conclusion and future directions

In this contribution, we explored how investigating different dimensions of moral cognition (i.e., decision‐making, judgment, inference) within the same experimental framework can facilitate a meaningful comparison of computational processes that may be common to multiple dimensions, and shed light on phenomena that emerge at the intersections of dimensions, such as moral hypocrisy, person‐centered moral judgments, and moral influence. These phenomena are ubiquitous in everyday moral life (cf. Graham, 2014; Hofmann et al., 2014) but research on their cognitive and neural mechanisms is still in its infancy.

We used a recently developed experimental framework (Crockett et al., 2014, 2017; Siegel et al., 2017, in press) to illustrate how this approach can illuminate the cognitive mechanisms of harm‐based moral cognition. We find preliminary support for our hypothesis that different dimensions of moral cognition are built upon basic utility computations that trade‐off costs and benefits to oneself against costs and benefits to others. This generic “utility calculus” (see also Jara‐Ettinger et al., 2016) accurately described moral decision‐making, judgment and inference processes, as well as their underlying neural correlates, across several experiments in the domain of harm (Table 1). Individual differences in subcomponents of utility, namely harm aversion, were correlated across dimensions of moral cognition.

**Table 1:**
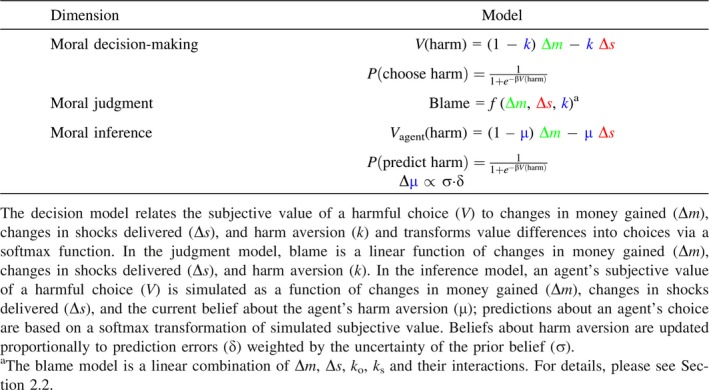
A common computational framework for measuring three dimensions of moral cognition

Although we have been focusing on harm‐based morality throughout this paper, we believe that this approach can be applied to investigate other moral domains, such as trust, loyalty, or purity. For example, using this approach to investigate decisions, judgments, and inferences about trust would involve measuring decisions in the trust game (King‐Casas et al., 2005; McCabe, Houser, Ryan, Smith, & Trouard, 2001; McCabe, Rigdon, & Smith, 2003) alongside moral judgments of others’ decisions in the trust game and predictions about whether others are likely to make trustworthy decisions (e.g. Behrens et al., 2008; Diaconescu et al., 2014). Using this approach to compare computational processes across multiple moral domains may help resolve the debate about the centrality of harm in moral cognition (Schein & Gray, 2015, Schein & Gray, 2018). Finally, we note that similar computational frameworks can be used to model cognitive processes that fall outside the domain of morality entirely, as we have shown in our moral inference work comparing learning about morality versus. competence (Siegel et al., in press).

There are a few notable limitations to the approach we propose here. First, examining multiple dimensions of moral cognition within the same paradigm may *force* computational processes to be shared across dimensions, when in reality different dimensions might employ rather different computations. Second, building paradigms that are amenable to computational modeling can require trading off real‐world richness for methodological rigor. Finally, identifying a computational model that provides a good fit to behavior or brain activity does not guarantee that the identified model is the *best* or most accurate model (Mars, Shea, Kolling, & Rushworth, 2012); an important and often overlooked aspect of computational cognitive modeling is falsifying candidate models in light of observed data (Palmintieri et al. 2017).

In spite of these limitations, computational frameworks may be especially useful in providing quantitative measures of individual differences in moral cognition that do not rely on self‐report, which can be less reliable in measuring traits that have a strong social desirability component. Such individual differences are also likely to be meaningful in the context of psychiatric disorders which often involve social difficulties (Mendez, 2009), providing biomarkers for intact and affected moral cognition complementary to traditional diagnosis. The model parameters could serve as an intermediate level (or “cognitive phenotype”; cf. Montague, Dolan, Friston, & Dayan, 2012) between biological and phenomenological descriptions of how a given (sub‐)clinical and psychiatric condition influences moral cognition and behavior, such as obsessive‐compulsive disorder (OCD; Harrison et al., 2012), psychopathy (Blair, 2007; Marsh et al., 2011), and personality disorder (Tyrer, Reed, & Crawford, 2015). This approach thus holds great promise not just for advancing our understanding of human morality, but also for reducing human suffering in health and disease.
